# A Case Report of Widely Disseminated Tuberculosis in Immunocompetent Adult Male

**DOI:** 10.5811/cpcem.2020.3.46183

**Published:** 2020-06-04

**Authors:** Samantha B. Esposito, Joseph Levi, Zachary M. Matuzsan, Alexandra M. Amaducci, David M. Richardson

**Affiliations:** Lehigh Valley Health Network/USF Morsani College of Medicine, Department of Emergency and Hospital Medicine, Allentown, Pennsylvania

**Keywords:** Disseminated tuberculosis, tuberculosis meningitis, miliary tuberculosis, immunocompetent adult, case report

## Abstract

**Introduction:**

Disseminated tuberculosis (TB) is rare, affects any organ system, and presents mainly in immunocompromised populations. Typical presentation is non-specific, posing a challenge for diagnosis.

**Case Report:**

This case presents an immunocompetent male presenting with severe headaches with meningeal signs. Lab and lumbar puncture results suggested bacterial meningitis, yet initial cerebral spinal fluid cultures and meningitis/encephalitis polymerase chain reaction were negative. A chest radiograph (CXR) provided the only evidence suggesting TB, leading to further tests showing dissemination to the brain, spinal cord, meninges, muscle, joint, and bone.

**Discussion:**

This case stands to acknowledge the difficulty of diagnosis in the emergency department (ED), and the need for emergency physicians to maintain a broad differential including disseminated TB as a possibility from the beginning of assessment. In this case, emergency physicians should be aware of predisposing factors of disseminated TB in patients presenting with non-specific symptoms. They should also acknowledge that TB may present atypically in patients with minimal predisposing factors, rendering the need to further investigate abnormal CXR images despite lab results inconsistent with TB.

**Conclusion:**

While this diagnosis is easily missed, early identification in the ED can lead to optimal treatment.

## INTRODUCTION

Tuberculosis (TB) stands as a global health problem caused by infection by *Mycobacterium tuberculosis*.^1^ From 1993 to 2018, there was a constant decrease in TB cases in the United States with a national incidence of 2.8 cases per 100,000 persons.^2^ At-risk populations include the elderly, immunocompromised individuals, the homeless, excessive alcohol use, and immigration from areas with high TB rates.^1^–^4^ Despite this low incidence, detection remains important because if not recognized or treated the infection can progress to disseminated TB, which can be fatal within a year.^5^–^6^

Disseminated, or miliary, TB is a progressive, life-threatening disease that results from lymphohematogenous dissemination of *M. tuberculosis* bacilli due to either primary dissemination or progression from years of untreated TB.^1^,^6^ Of all TB cases, only 1–2% are disseminated TB.^1^,^4^ Although rare, predisposing factors to disseminated TB include elderly patients, individuals with childhood infections, human immunodeficiency virus (HIV), alcohol abuse, diabetes, chronic liver or kidney failure, organ transplant, pharmacological immunosuppressants, pregnancy, and symptoms lasting longer than 12 weeks.^1^,^3^,^7^

TB can disseminate to any organ system of the body, 22% of which disseminate to the central nervous system (CNS) including meningitis, cerebral tuberculoma, tuberculoma abscess, and thoracic transverse myelopathy.^1^,^6^ TB meningitis accounts for 10–30% of CNS disseminations.^1^ Because of disseminated TB’s nonspecific clinical presentation such as weight loss, night sweats, cough, fever, anorexia, and weakness, it is increasingly difficult to diagnose despite the urgent need.^4^–^5^,^7^

Because TB with dissemination to the CNS is rarely seen in the emergency department (ED), the current case stands to acknowledge the importance of early identification in the ED leading to optimal management and treatment. We report the case of an immunocompetent adult male presenting to the ED with severe headaches due to disseminated TB to the brain, spinal cord, meninges, muscle, joint, and bone.

## CASE REPORT

A 30-year old male, who immigrated to the United States from Mexico three years prior, presented to the ED with complaints of a headache, weight loss, and vomiting. The patient reported the headache was localized to his bitemporal area without radiation, and progressively worsened over the two weeks prior to his arrival for evaluation. He also reported neck pain and stiffness associated with the headache. His neck pain increased with flexion and extension, and acetaminophen and nonsteroidal anti-inflammatory drugs did not provide relief. Additionally, the patient denied previous similar headaches, cough, congestion, sore throat, chest pain, shortness of breath, abdominal pain, back/hip pain, or changes in bowels or bladder.

His initial temperature was 37.5° Celsius. On exam he was noted to have an ill and sickly appearance. He had neck rigidity and decreased range of motion. Brudzinski’s sign was noted; however, Kernig’s sign was not present. The rest of the exam was normal.

**Image 1:**
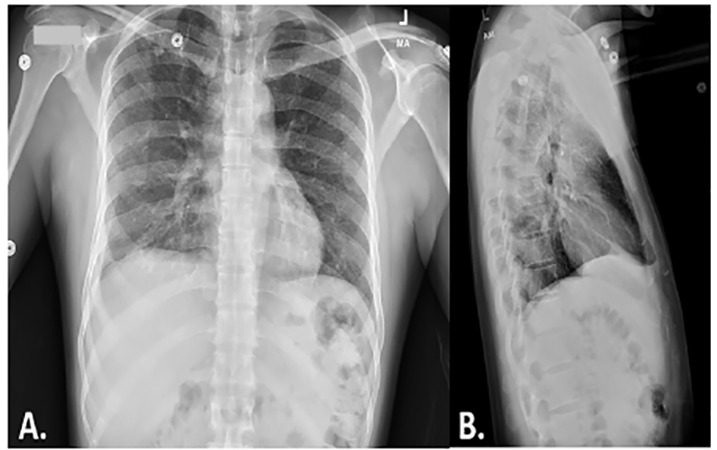
Initial chest radiograph with reticulonodular diffuse lung pattern that can be seen in infection. A) anteroposterior view. B) lateral view.

Complete blood count, comprehensive metabolic panel, chest radiograph (CXR), head computed tomography (CT), and lumbar puncture (LP) were performed. The patient’s labs revealed a white blood count of 4.0 per high power field, mild hyponatremia, but were otherwise unremarkable. His initial head CT revealed no acute abnormalities. However, the CXR showed reticulonodular diffuse lung pattern as typically seen with infection (Image 1). LP results (neutrophils 91% (normal 0–2%), protein 351 milligrams per deciliter (mg/dL) (normal 15–45 mg/dL), and glucose 14 mg/dL (normal 40–70 mg/dL) were concerning for bacterial meningitis; therefore, ceftriaxone and vancomycin were administered. The patient was then admitted for further evaluation and management pending further cerebral spinal fluid (CSF) study results.

**CPC-EM Capsule**

What do we already know about this clinical entity?

Disseminated tuberculosis (TB) is a rare progression of TB affecting many at risk populations, such as the immunocompromised and immigrants from areas with high TB rates.

What makes this presentation of disease reportable?

Disseminated TB to the meninges, spinal cord, bone, joint, and muscle is exceedingly rare, and it is important to have an early clinical suspicion in the emergency department to prevent delay in diagnosis and treatment.

What is the major learning point?

This case demonstrates that widely disseminated TB can present with vague, nonspecific symptoms with minimal risk factors and limited diagnostic evidence.

How might this improve emergency medicine practice?

This may improve emergency medicine practice by increasing awareness of TB and perhaps lower threshold for early testing for TB by serum or lumbar puncture analysis to prevent delay of diagnosis and treatment.

**Image 2:**
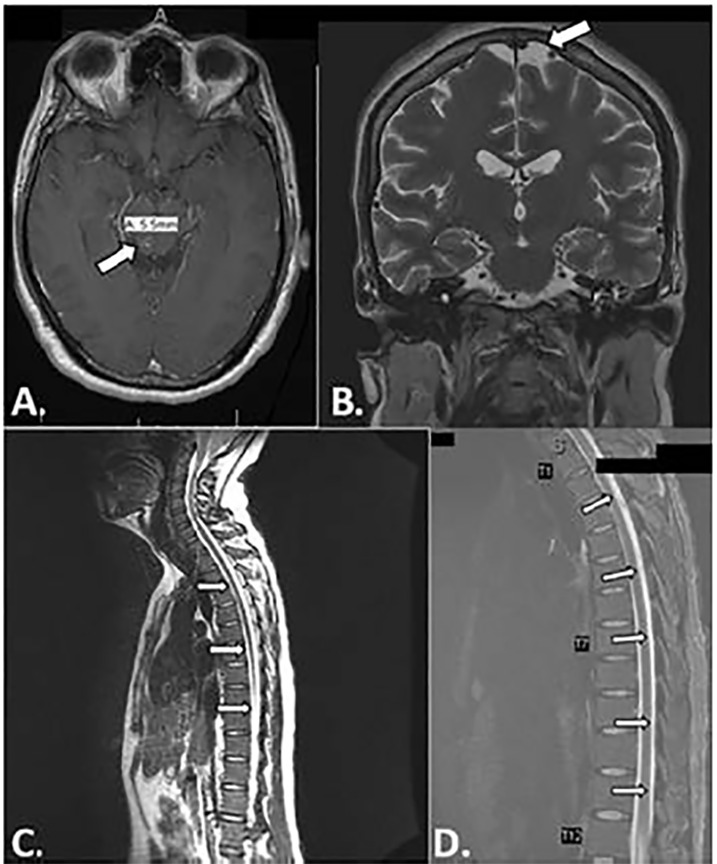
Magnetic resonance imaging of the brain, cervical spine and thoracic spine: A) axial view of the brain showing 5.5 millimeter (mm) small ring-shaped enhancement in the posterior right side of the midbrain potentially indicating infection; B) coronal view of the brain showing 7.4 mm incidental arachnoid cyst of the left frontal lobe; C) sagittal view of the cervical and thoracic spine showing smooth enhancement along the pleural surface of the thoracic spinal cord; D) magnified sagittal view of the thoracic spine showing enhancement along leptomeninges potentially indicating meningitis.

**Image 3:**
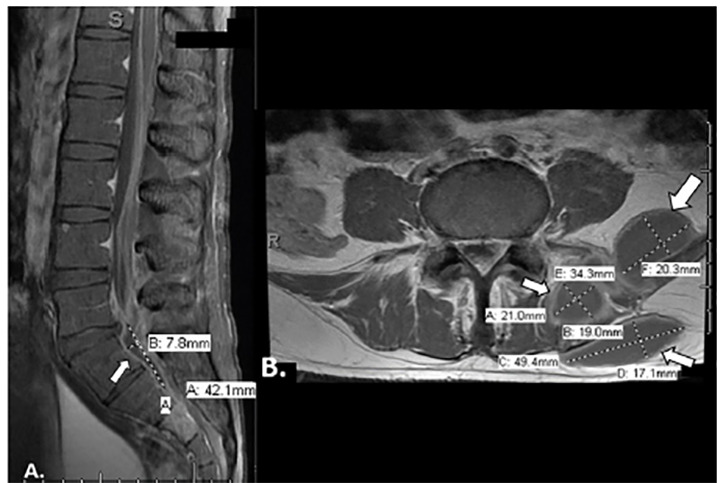
Magnetic resonance imaging of the lumbar spine: A) sagittal view showing an epidural abscess measuring approximately 0.3 × 0.8 × 4.2 centimeters causing severe compression of the thecal sac (white arrows); B) axial view showing a paraspinal abscess at the fourth and fifth lumbar level contiguous with component extending to the subcutaneous soft tissue, and an additional abscess extending inferiorly into the left sacral paraspinal musculature (white arrows).

An infectious disease physician (ID) was consulted by the hospital medicine team given concern for bacterial meningitis and worsening clinical symptoms. Initial cultures, meningitis/encephalitis CSF polymerase chain reaction (PCR), and HIV test results were negative, and so antibiotics were narrowed. On hospital day (HD) four, magnetic resonance imaging (MRI) was performed as no bacteria was yet identified to explain the abnormal CSF results. Brain and spine MRI showed multiple ring-enhancing foci in the brain parenchyma and diffuse enhancement along the thoracic spinal cord consistent with leptomeningitis (Image 2). Additionally, the MRI showed a large paraspinous abscess, a left epidural abscess compressing the thecal sac, and an abnormal signal enhancement in the left sacrum indicating sacral osteomyelitis with septic arthritis of the left sacroiliac joint (Image 3). However, they were again broadened given MRI findings. Following these results, ID recommended repeating the LP to specifically test for acid-fast bacilli (AFB) cultures and smear to determine whether mycobacterium was the source of the infection. Fluoroscopy-guided incision and drainage (I&D) of left paraspinal abscess drainage was also performed by interventional radiology to determine the source of the paraspinal abscess.

On HD seven, the cultures from both the LP and abscess drainage were positive for *M. tuberculosis* complex (MTC). The patient was diagnosed with disseminated TB to the meninges and spinal cord, with paraspinal abscesses, sacral osteomyelitis, and sacroiliac septic arthritis. A high sensitivity chest, abdomen, and pelvic CT confirmed this diagnosis as there was no evidence of active disease, but rather signs of disseminated TB. The patient was treated with rifampicin, isoniazid, pyrazinamide, and ethambutol (RIPE) therapy, pyridoxine, and dexamethasone taper, and he was confined to a negative pressure room until three negative sputum cultures were obtained. Vancomycin and cefepime were discontinued. On HD 12, per orthopedic surgery, an I&D of paraspinal abscesses and left sacroiliac joint was successfully completed. The specimen from this surgery grew AFB; therefore, the patient’s RIPE therapy and steroids were continued. On HD 27, the patient was hemodynamically stable and discharged with RIPE, pyridoxine, and steroid prescriptions, and instructed to follow up with the Department of Health and Infectious Disease. The Department of Health was aware of the case and following it through the patient’s hospital stay and follow-up.

## DISCUSSION

This case presents a healthy 30-year-old male diagnosed with TB disseminated to the CNS, and highlights the need for early and accurate identification of disseminated TB for optimal patient outcomes. Despite previous reports of CNS dissemination of TB, this case stands to acknowledge the difficulty of diagnosis in the ED, and the need for emergency physicians (EP) to maintain a broad differential including disseminated TB as a possibility from the beginning of assessment. 8–12 In this case EPs should be aware of predisposing factors of disseminated TB in patients presenting with non-specific symptoms. These predisposing factors include impaired cell-mediated immunity as seen in HIV/acquired immune deficiency syndrome patients, increased use of immunosuppressive drugs, diminished ability of the liver to clear bacteria from the bloodstream as seen in advanced liver disease among others, and recent immigration from areas with high rates of TB.^1^,^2^,^7^ Additionally, they should acknowledge that TB may present atypically in patients with minimal predisposing factors, rendering the need to further investigate abnormal CXR images despite laboratory results inconsistent with TB. EPs should also have a low threshold to order an AFB culture for the first CSF analysis with any suspicion for TB in a patient.

CXRs are pertinent for TB diagnosis by demonstrating discrete, uniform, pulmonary opacities measuring less than two millimeters in diameter.^5^ This was the only indication of TB in the present case, which expanded the differential diagnosis to include TB through AFB with CSF analysis to confirm TB. This led to a prompt diagnosis and management of the current patient. Although radiographs can be beneficial for TB diagnosis, approximately half of disseminated TB cases do not present with this typical lung pattern, rendering alternate means of imaging such as high-resolution CT or MRI.^1^,^4^,^7^ Despite robust therapeutic options, mortality rates are high as early diagnosis is hard to obtain because there is a lack of a gold standard for diagnosis.^5^ Currently, diagnosis requires presence of diffuse miliary infiltrate on CXR or high-resolution CT. Confirmation occurs with other methods such as isolation and PCR of sputum, body fluids, or biopsy specimen.^1^ However, these confirmation methods may not produce positive results until late in the disease progression.^1^

It has been suggested that close examination of organ systems can help determine TB dissemination.^5^ For example, in the present case the patient presented with signs of meningitis as well as hyponatremia, which serve as indications of TB meningitis.^5^ Contrast-enhanced, high-resolution CT and MRI of the brain and spine may be of increased use for TB meningitis as they can show the disease progression and miliary pattern.^1^,^4^–^5^ These additional images in the current case confirmed the dissemination of the disease to the meninges, paraspinal abscess, sacral osteomyelitis, and sacroiliac joint septic arthritis, which enabled further treatment for these specific disseminations.

Treatment regimens for disseminated TB vary in duration and require careful evaluation of the organ systems involved, especially in TB meningitis.^4^ Typical treatments for TB meningitis include RIPE therapy as initial treatment for two months, followed by 7–10 months of isoniazid and rifampicin therapy alone.^13^ Follow up of disseminated TB patients has showed 52% of patients improve with this treatment.^6^ Longer treatment duration occurs more often in men and dissemination with bone/joint involvement, which may also require surgery.^4^,^13^ Additionally, treating the inflammatory response in mycobacterial meningitis has been seen to improve outcomes by reducing CSF inflammation.^13^ Therefore, dexamethasone tapers for 6–8 weeks are also recommended.^13^–^14^ Repeat LP should be used to monitor changes in CSF throughout treatment.^13^

## CONCLUSION

Disseminating TB to the meninges, spinal cord, bone, joint, and muscle is exceedingly rare, and it is important to diagnose early in the ED. This case serves to demonstrate that TB disseminations can present with complaints typical of meningitis with the only indications suggesting TB being a military pattern on CXR, which needs to be further investigated despite limited risk factors and laboratory results not indicating TB. Additionally, this case shows successful treatment and outcome for the patient with early diagnosis and treatment management.
